# Interaction between hTIM-1 and Envelope Protein Is Important for JEV Infection

**DOI:** 10.3390/v15071589

**Published:** 2023-07-21

**Authors:** Zhenjie Liang, Junhui Pan, Shengda Xie, Xingmiao Yang, Ruibing Cao

**Affiliations:** MOE Joint International Research Laboratory of Animal Health and Food Safety, College of Veterinary Medicine, Nanjing Agricultural University, Nanjing 210095, China; 2019207022@stu.njau.edu.cn (Z.L.); 2018207041@njau.edu.cn (J.P.); 2018107035@njau.edu.cn (S.X.); 2020207038@stu.njau.edu.cn (X.Y.)

**Keywords:** Japanese encephalitis virus, envelope protein, hTIM-1, interaction

## Abstract

Japanese encephalitis virus (JEV), a mosquito-borne zoonotic virus, is one of the most important causes of human viral encephalitis. JEV relies on various attachment or entry co-factors to enter host cells. Among these co-factors, hTIM-1 has been identified as an attachment factor to promote JEV infection through interacting with phosphatidylserine (PS) on the viral envelope. However, the reasons why JEV prefers to use hTIM-1 over other PS binding receptors are unknown. Here, we demonstrated that hTIM-1 can directly interact with JEV E protein. The interaction between hTIM-1 and JEV relies on specific binding sites, respectively, ND114115 in the hTIM-1 IgV domain and K38 of the E protein. Furthermore, during the early stage of infection, hTIM-1 and JEV are co-internalized into cells and transported into early and late endosomes. Additionally, we found that the hTIM-1 soluble ectodomain protein effectively inhibits JEV infection in vitro. Moreover, hTIM-1-specific antibodies have been shown to downregulate JEV infectivity in cells. Taken together, these findings suggested that hTIM-1 protein directly interacts with JEV E protein and mediates JEV infection, in addition to the PS-TIM-1 interaction.

## 1. Introduction

Japanese encephalitis virus (JEV) is one of the most important causes of human viral encephalitis, mainly prevalent in eastern and southern Asia [1]. The World Health Organization (WHO) estimates that there are approximately 68,000 cases of Japanese encephalitis (JE) worldwide each year, resulting in approximately 30% mortality [2,3]. JEV belongs to the Flavivirus genus of the Flaviviridae family, which also includes dengue virus (DENV), West Nile virus (WNV), Zika virus (ZIKV), and others [4,5]. JEV circulates in a natural cycle involving a variety of animal species, including Culex mosquitoes, wild wading birds, and pigs, which serve as natural and amplified hosts. Humans and horses are considered dead-end hosts for JEV infection [6,7]. JEV is an enveloped RNA virus with a single-stranded, positive-sense RNA genome of ~11 kb in length. Its genome encodes a single polyprotein, which is cleaved into three structural proteins (C, prM, and E) and seven nonstructural proteins (NS1, NS2A, NS2B, NS3, NS4A, NS4B, and NS5) by host and virus-encoded proteases [8,9,10].

JEV can infect a wide range of cells, including mammalian, avian, and mosquito, by using different attachment factors or receptors to enter cells [11]. Heat shock proteins (HSPs) mainly include GRP78/HSP70/90, which promote JEV entry into different cells. HSP70 is considered to be the putative receptor for JEV entering into Neuro2a cells [12]. Simultaneously, the association between HSP70 and lipid rafts promotes JEV infection in Huh7 cells [13]. GRP78, a component of numerous cell membranes, has also been identified to facilitate JEV entry and replication in recent years [14]. As a type of skeleton protein, vimentin has been shown to interact with E protein to promote JEV entering into PK-15 cells [15]. C-type lectins including DC-SIGN and L-SIGN mainly promote JEV attachment and internalization by interacting with carbohydrates on the E and prM proteins [16]. Integrin αVβ3 has also been proved to mediate JEV adsorption and entry [17].

The hTIM-1 protein is a member of T cell immunoglobulin and mucin domain (TIM) family and mainly expressed on T cells and a broad range of epithelial cells [18,19]. hTIM-1 is a type I cell-surface glycoprotein with an immunoglobulin V-like domain (IgVD), a mucin-like domain (MD), a single transmembrane region, and a cytoplasmic tail domain. The characteristic CC’ loop and the hydrophobic FG loop of TIM-1 IgVD shapes a narrow cavity where the acidic phosphate group of PS penetrates and co-ordinates to a metal ion bound to conserved residues (ND114115) in the TIM proteins. The cavity is stabilized by some conserved residues of the IgVD bound with PS or lipid bilayer, as well as the conformation of disulfide bonds [20,21]. This important phosphatidylserine (PS)-binding site regulates many physiological or immune-related processes [21]. Envelope viruses incorporate PS into the viral membrane and hijack the process, known as “apoptotic mimicry”, to bind to the cell surface and facilitate viral entry [22,23].

The significance of hTIM proteins in virology was initially recognized in their ability to enhance viral entry. TIM proteins were originally recognized as “receptors” for the non-enveloped hepatitis A virus (HAV) [24,25]. Previous studies have demonstrated that hTIM-1, with some other but not all PS receptors, serves as an attachment or entry factor and promotes multiple envelope viruses’ entry, including the flavivirus [26,27], filovirus, baculovirus [28], alphavirus [29], and others, through direct interaction with PS on the viral envelope. However, recent research has revealed that hTIM-1 is preferentially used by filovirus and flaviviruses compared to other PS receptors [26,27,30]. hTIM-1 protein ubiquitination promotes DENV entry and it has been identified as a first bona fide receptor [31]. hTIM-1 has also been found to directly interact with Ebolavirus (EBOV) glycoprotein (GP) to promote its infection. hTIM-1 serves as a dual-attachment receptor for EBOV by interacting directly with viral GP and the PS on the viral envelope [30]. Ubiquitination of ZIKV E protein promotes viral entry, which is associated with the interaction between the envelope protein and hTIM-1 [32]. These findings raise a crucial question on whether hTIM-1 functions as an attachment factor or also can interact with envelope protein to promote viral infection.

The JEV envelope protein is typically found as a dimer and consists of three independent structural domains I, II, and III (EDI, EDII, and EDIII), which mediate virus receptor binding, internalization, and virus fusion with host cell membranes or endosomes [33,34,35]. The amino acid residue K38, highly conserved in the E protein of flaviviruses, can be ubiquitinated by TRIM7 and interacts with hTIM-1 to promote ZIKV entry. However, substitution of the amino acid K with R at position 38 weakens the interaction between hTIM-1 and ZIKV E protein [32].

Here, we showed that hTIM-1 directly interacts with JEV E protein, in which the PS binding sites of hTIM-1 and K38 in E protein play an important role. Additionally, JEV and hTIM-1 are both internalized into cells and together transported to early and late endosomes. The hTIM-1 ectodomain soluble protein inhibits JEV infection and entry. hTIM-1 antibody also impedes JEV infection. Altogether, our findings suggest that the interaction between hTIM-1 and E protein mediates JEV infection.

## 2. Materials and Methods

### 2.1. Cells and Viruses

Lung adenocarcinoma cell line (A549) and Aedes albopictus cell line (C6/36) were kept in our lab and grown in RPMI 1640 medium (31870082, GIBCO, Invitrogen, Carlsbad, CA, USA) with 10% fetal bovine serum (FBS, FSP500, Excell, Shanghai, China), 100 μg/mL streptomycin, and 100 IU/mL penicillin (P1400, Solarbio, Beijing, China). Human embryonic kidney cells (HEK-293T) and baby hamster kidney cells (BHK-21) were maintained in Dulbecco’s modified Eagle’s medium (DMEM) (11965092, GIBCO, Invitrogen, Carlsbad, CA, USA), supplemented with 10% FBS, 100 μg/mL streptomycin, and 100 IU/mL penicillin at 37 °C with 5% CO_2_. The JEV strains NJ2008 (GenBank: GQ918133.2), HEN0701 (GenBank: FJ495189.1), and SA14-14-2 (GenBank: MK585066.1) were kept in our lab and were propagated and titrated on cells by plaque assays.

### 2.2. Plasmids and Antibodies

The pcDNA3.1(−)-TIM-1 and pVAX1-NS1′ were preserved in our laboratory. JEV E gene was inserted into p3×FLAG-CMV-7.1. hTIM-D114A-His, hTIM-N115A-His, and hTIM-MD were cloned into pcDNA3.1 (+). hTIM-IgVD was cloned into pCAGGS. hTIM-ECD was inserted into prokaryotic expression vectors pET-28a (+) and pGEX. Mouse anti E monoclonal antibody was kept by our lab. Mouse anti-human TIM-1 monoclonal antibody (MAB1750, R&D systems, Minneapolis, MN, USA) and goat anti-TIM-1 polyclonal antibody (AF1750, R&D systems, Minneapolis, MN, USA) were used for detecting the TIM-1 protein in Western blot and IFA. Mouse anti-His antibody (66005-1-Ig, Proteintech, Rosemont, IL, USA) was used for the enrichment of TIM-1-His protein. Mouse anti-GAPDH polyclonal antibody (sc-25778, Santa Cruz, Dallas, TX, USA) was used for Western blot. Rabbit anti-JEV E polyclonal antibody (GTX125867, GeneTex, Irvine, CA, USA), rabbit anti-JEV NS3 polyclonal antibody (GTX125868, GeneTex, Irvine, CA, USA), and rabbit anti-JEV polyclonal prM antibody (GTX131833, GeneTex, Irvine, CA, USA) were used to detected the JEV E, NS3 and prM protein, respectively, in Western blot analyses. Donkey anti-goat IgG-Alexa Fluor488 (ab150129, Abcam, Cambridge, UK), donkey anti-rabbit IgG Alexa Flour 594 (ab150076, Abcam, Cambridge, UK), and donkey anti-mouse Alexa Flour647 (ab150107, Abcam, Cambridge, UK) were used for IFA.

### 2.3. Western Blot

Cells were washed with cold PBS three times and lysed using RIPA buffer (89900, ThermoFisher, Waltham, MA, USA) for 15 min at 4 °C. Cell lysates were clarified by centrifugation at 12,000× *g* for 15 min (5424R, Eppendorf, Hamburg, Germany). The centrifugal supernatant was collected and separated by SDS PAGE, which were then transferred into PDVF membranes (1620177, Bio-Rad, Hercules, CA, USA) in a Trans-Blot^®^ Turbo Transfer System (VE-586, Tanon, Shanghai, China). Afterwards, the membranes were blocked with 5% skimmed milk (BS102, Biosharp, Shanghai, China) for 1.5 h at room temperature (RT). Primary antibodies were incubated overnight at 4 °C. Secondary antibodies were used to detect the expression of proteins.

### 2.4. Co-Immunoprecipitation (Co-IP) Assays

The cells were lysed in NP-40 lysis buffer (P0013F, Beyotime, Shanghai, China) for 30 min at 4 °C and were clarified by centrifugation at 10,000× *g* for 15 min at 4 °C to remove cell debris. Then, 80 μL of the supernatant was removed for input sample and the remaining lysate was incubated with 1 μL of the appropriate control mouse IgG (A7028, Beyotime, Shanghai, China) and 20 μL proteinA/G PLUS-Agarose (Santa-Cruz, Dallas, TX, USA) overnight at 4 °C on a flip shaker. Then, the agarose beads were removed by centrifugation and the supernatant was collected and incubated with anti-His antibodies (Proteintech, Rosemont, IL, USA) or TIM-1 polyclonal antibodies for 8 h at 4 °C. Protein A/G PLUS-agarose was then added to the supernatant for about 6 h with a shaker. After conjugation, the beads were washed 4 times with prechilled NP-40 lysis buffer. The agarose beads were resuspended into PBS and 5× loading buffer for Western blotting analysis.

### 2.5. GST Pull-Down Assays

The N-terminal GST-tagged TIM-1 protein ectodomain soluble protein (amino acids, 21-296) or control pGEX was expressed in 15 mL *E. coli* and sonicated to obtain the translucent supernatant, respectively. The supernatant was incubated with GST magnetic beads for 4 h at 4 °C with a flip shaker. Then, the beads were rinsed with PBS three times, the lysates added with E protein, and incubated for 8 h at 4 °C with rotation. The beads were washed three times again and the target protein eluted using elution buffer (50 mM Tris-HCl, 10 mM–20 mM Reduced glutathione, pH 8.0) and were subjected to Western blot.

### 2.6. Plaque Assay

BHK-21 cells were kept by our lab and seeded in 6-well plates and incubated at 37 °C with 5% CO_2_. Cells were inoculated with JEV in a 10-fold serial dilution using serum-free DMEM and incubated at 37 °C with 5% CO_2_ for 1.5 h, while the untreated cells were regarded as a negative control. After removal of virus suspension, the cells were overlaid with a 1:1 mixture of 2% low-melting agarose (A8350, Solarbio, Beijing, China) and 2-fold DMEM including 4% FBS. The cells were stained with crystal violet (BL802A, Biosharp, Hefei, China) for 4 h after the plaque appeared on the plate. The titer of the virus was determined by counting the number of virus plaques.

### 2.7. Quantitative Real-Time Polymerase Chain Reaction (qPCR)

The total RNA in cells was extracted using TRIZOL reagent (9108, TaKaRa, Kyoto, Japan) and was reverse-transcribed into cDNA using HiScript ⅢRT SuperMix (R323, Vazyme, Nanjing, China). qPCR was performed by ChamQ SYBR-qPCR Master Mix (Q321, Vazyme, Nanjing, China) with gene-specific primer pairs (Table 1). The quantification of the gene expression was calculated according to the 2^–ΔΔCt^ method [36].

### 2.8. Immunofluorescence Assays (IFA) or Confocal Fluorescence Microscopy

Cells were transfected with plasmids or infected with JEV, while the cells transfected with empty vector or untreated cells were analyzed as negative control, respectively. After 24 to 48 h, the cells were washed with PBS and fixed with 4% paraformaldehyde (BL539A, Biosharp, Shanghai, China) at RT for 15 min. Then, cells were permeabilized with 0.1% Triton X-100 (P0096, Beyotime, Shanghai, China) at RT for 10 min. The cells were incubated with the primary antibody at 4 °C overnight. The next day after washing, the cells were incubated with mixed second antibodies at 37 °C for 45 min. Finally, nuclei were stained using 4,6-diamidino-2-phenylindole (DAPI, KGA215-50, KeyGen Biotech, Nanjing, China) for 5 min. Images were captured and analyzed by fluorescence microscopy (Axio Vert. A1, ZEISS) or confocal microscopy (A1, NIKON).

### 2.9. Protein Neutralization Infection or Entry Assays

The N-terminal and C-terminal His-tagged soluble ectodomain of hTIM-1 (amino acids, 21-296) was expressed and purified by HisTrap HP (17524701, GE, Boston, MA, USA). HEK293T cells and A549 cells were grown on 96-well plates. JEV strain NJ2008 at MOI = 0.5 was incubated with different concentrations of TIM-1 ECD protein ranging from 0 μg/mL to 100 μg/mL at 4 °C for 1 h before being used to infect the cells. The virus–protein mixtures were added into the cells and incubated at 37 °C for 2 h and changed into 2% FBS fresh medium. At 36 h post-infection, the cells were fixed with 4% paraformaldehyde and subjected to IFA by using mouse anti-JEV E antibody. The relative infection ratio was calculated by using software. For entry neutralization assays, the 293T cells and A549 cells were seeded into 24-well plates and infected with mixtures including JEV strain NJ2008 (MOI = 10) and TIM-1 protein with different concentrations for 1 h. Then, the supernatant was removed and transferred to 37 °C for another 45 min. Then, the total RNA in cells was extracted and subjected to qPCR. The results were then analyzed using GraphPad Prism.

### 2.10. Antibodies Neutralization Assays

The polyclonal antibodies were obtained from immunized mice and purified with MAbTrapTM Kit (17-1128-01, GE, Boston, MA, USA). In addition, the commercial mouse IgG (A7028, Beyotime, Shanghai, China) was used as an isotype control. The cells were seeded into 96-well plates and incubated with 30 μL of medium containing different concentrations of antibodies and isotype antibody at 4 °C for 1 h. Then, the cells were inoculated with JEV strain NJ2008 (MOI = 0.5) at 37 °C for another 1h and changed into 2% FBS fresh medium. After 36 h infection, the cells were fixed with formaldehyde and subjected to IFA. The infection ratios were determined by using the ImageJ software.

### 2.11. Statistical Analysis

Graphical representation and statistical analyses were performed using GraphPad Prism 6 software. A *t*-test was used to compare the data from the pairs of treated and untreated groups. Results are shown as means ± SD from three independent experiments. Statistical significance is indicated by asterisks (* *p* < 0.05; ** *p* < 0.01) in the figures.

## 3. Results

### 3.1. hTIM-1 Directly Interacts with JEV E Protein

**Figure 1:**
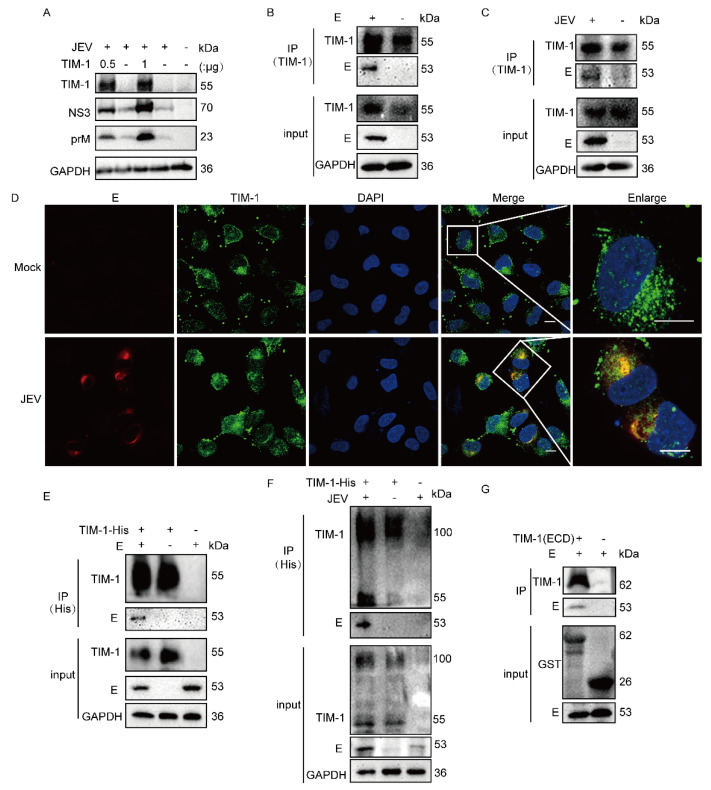
TIM-1 directly interacts with JEV E protein: (**A**) 293T cells were transfected with pTIM-1 at different doses or vector for 24 h and then infected with JEV NJ2008 for 36hpi. The cell samples were subjected to Western blot. (**B**,**C**) A549 cells were transfected with E plasmids or vector for 36 hpi (**B**); A549 cells were infected with JEV strain NJ2008 (MOI = 1) for 36 hpi (**C**), then the cells were harvested and subjected to Co-IP assays via using mouse anti-TIM-1 antibody for enriching endogenous TIM-1. Input and IP assays were then subjected to Western blot by using goat anti-TIM-1 and rabbit anti-E antibodies, along with GAPDH as a loading control. (**D**) A549 were infected with NJ2008 (MOI = 0.1) for 36hpi, then fixed with 4% paraformaldehyde and subjected to immunofluorescence by using rabbit anti-E antibody (red) and goat anti-TIM-1 antibody (green). The nuclei were stained with DAPI. Scale bars, 10 μm. (**E**,**F**) 293T cells were co-transfected with TIM-His and E plasmids or vector for 36 h (**E**); 293T were transfected with TIM-His for 24 hpi and then infected with NJ2008 (MOI = 1) for 36 hpi (**F**). Then, the cells were harvested for Co-IP assays. (**G**) GST-TIM (ECD) protein was expressed in *E. coli* and pulled down E protein from cell lysates and then subjected to Western blotting analysis.

The hTIM-1 protein promotes JEV infection in 293T cells (Figure 1A). To explore the potential interactions between TIM-1 and JEV envelope protein in vitro in addition to the PS-TIM-1 interaction, co-immunoprecipitation (Co-IP) assays were conducted in cells. A549 cells endogenously expressing hTIM-1 protein were either transfected with E plasmid or infected with JEV strain NJ2008. The results indicated that hTIM-1 protein interacted with JEV E protein (Figure 1B,C). Meanwhile, the confocal results showed that E protein colocalized with hTIM-1 in A549 cells (Figure 1D). In 293T cells, the results also showed that exogenously expressing TIM-1 protein interacted with E protein whenever the cells were infected with JEV or overexpressing E protein (Figure 1E,F). To further verify whether hTIM-1 directly interacted with JEV E, a GST pull-down assay was performed using recombinant GST-tagged hTIM-1 protein and the lysates of 293T cells transfected with E plasmid. The results indicated that hTIM-1 protein directly interacted with JEV E protein (Figure 1G). Collectively, these findings suggested that hTIM-1 protein specificly interacted with JEV E protein.

### 3.2. N114 and D115 of hTIM-1 and K38 of JEV E Are Crucial for Their Interaction

**Figure 2:**
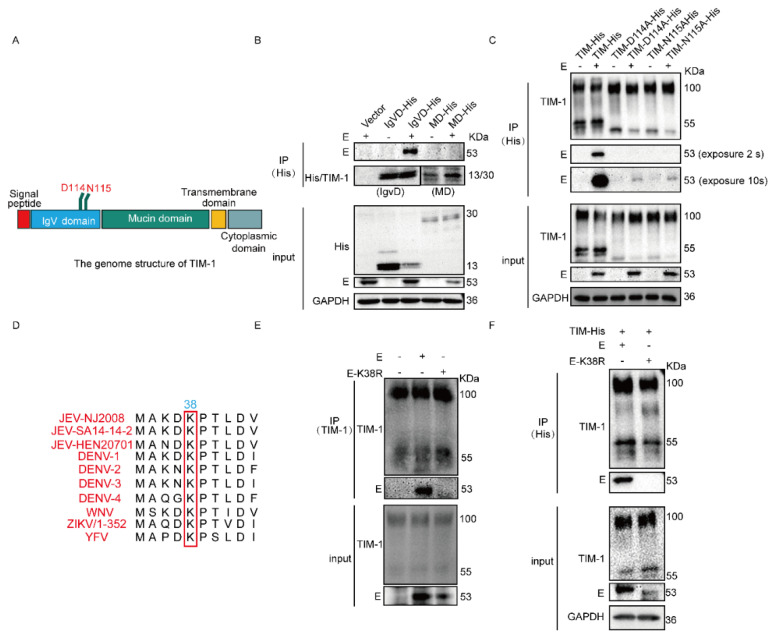
ND114115 of TIM-1 and K38 of E are crucial for the interaction between TIM-1 and E protein: (**A**) schematic diagram of the gene structure of TIM-1 protein. (**B**) 293T cells were co-transfected with TIM-1 truncations plasmids (IgVD and MD), E, or vector for 36 h. The cell samples were collected and subjected to Co-IP assays. The anti-His antibody was used to enrich the TIM-1 protein. (**C**) 293T cells were co-transfected with TIM-1 mutant plasmids (TIM-N114A-His and TIM-D115A-His) and E or vector plasmids for 36 h and subjected to Co-IP assays. (**D**) The comparation of partial E sequences of different flaviviruses. (**E**) A549 cells were transfected with E and its mutation E(K38R) plasmid for 36 h and then the cellular lysates were subjected to Co-IP assays. The TIM-1 protein was enriched via mouse TIM-1 antibody. (**F**) 293T cells were transfected with TIM-1-His and E or E(K38R) plasmid, respectively. The cell lysates were harvested after 36 h and subjected to Co-IP assays.

To further confirm which domain of hTIM-1 interacts with JEV E protein, we co-transfected with plasmids expressing IgVD or MD and E into 293T cells, respectively (Figure 2A). Co-IP results showed that only the IgVD interacted with E protein (Figure 2B). IgVD domain contains the most important sites N114 and D115 [21]. So, we predominantly tested whether these two sites contributed to the interaction between hTIM-1 and JEV E protein. TIM-N114A-His and TIM-D115A-His plasmids were co-transfected with JEV E plasmid into 293T cells, respectively. The results showed that the mutations at N114 and D115 of hTIM-1 markedly dampen the interaction with E protein. However, after the mutation of PS binding sites, the weak interaction still exists, indicating that other amino acid residues in the TIM-1 protein may play a minor role in their interaction (Figure 2C). The amino acid residue K38 of E protein in ZIKV plays the key role to interacts with TIM-1 and drives virus entry and pathogenesis [32]. The residue was highly conserved among flaviviruses (Figure 2D). Therefore, the plasmids encoding E or its mutant E(K38R) were transfected into A549 cells, respectively. The results indicated that the interaction between E and hTIM-1 protein was severely impaired when the lysine at the position of 38 was mutated into arginine (Figure 2E). Similar results were obtained by using HEK-293T cells (Figure 2F). Collectively, we concluded that the N114 and D115 of TIM-1 protein and K38 site of JEV E protein were important for their interaction.

### 3.3. JEV and hTIM-1 Are Internalized into Cells and Transported Together in Early and Late Endosomes

**Figure 3:**
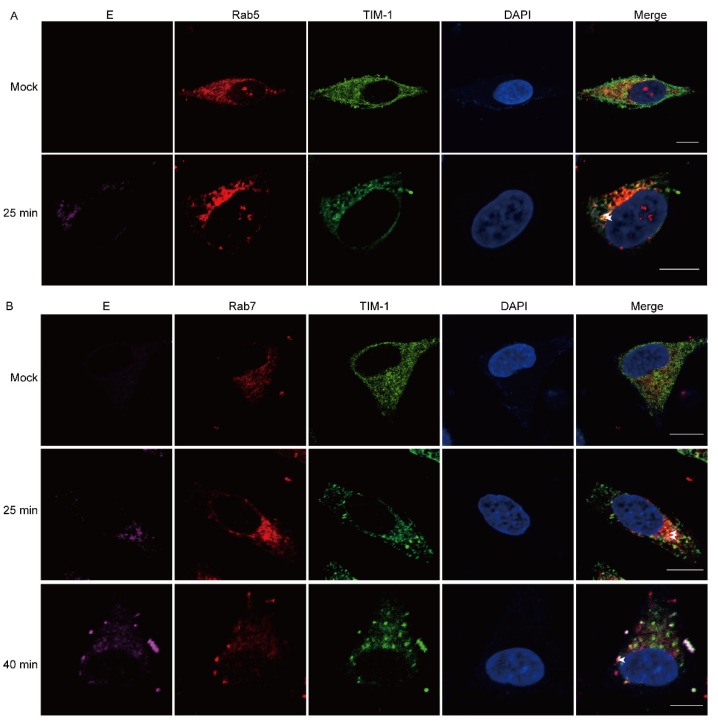
JEV and TIM-1 are internalized into cells and transported into early and late endosomes (**A**,**B**) A549 cells were infected with JEV (MOI = 10) at 4 °C for 1 hpi, then shifted to 37 °C for indicated time points, respectively. Cells were fixed and subjected to immunofluorescence by using mouse anti-JEV E monoclonal antibody, goat anti-TIM-1 polyclonal antibody, and rabbit anti-Rab5 (**A**); -Rab7 (**B**); the nuclei were stained with DAPI. The white arrow indicates the colocalized protein. Scale bars, 10 μm.

Since hTIM-1 protein can interact with JEV E protein to mediate JEV entering cells. We investigated whether hTIM-1 assisted in JEV intracellular transport. We examined the subcellular colocalization of the E protein, hTIM-1 protein, Rab5 (early endosome marker), and Rab7 (late endosome marker) protein in JEV NJ2008-infected A549 cells using immunofluorescence staining assays. The results showed that Rab5 could colocalize with hTIM-1 and JEV E protein at 5 min post-infection (mpi) (Figure 3A), whereas Rab7 colocalized with them at 25 mpi and 40 mpi (Figure 3B). Therefore, these results suggested that hTIM-1 protein co-operated with viral transportation within the endosomes.

### 3.4. Recombinant hTIM-1 Ectodomain Protein Can Inhibit JEV Infection in Cells

**Figure 4:**
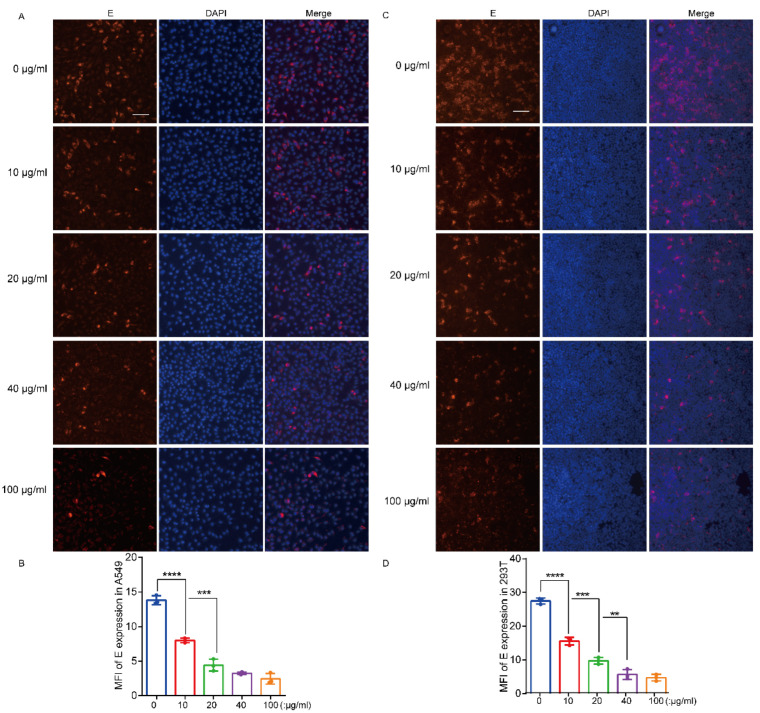

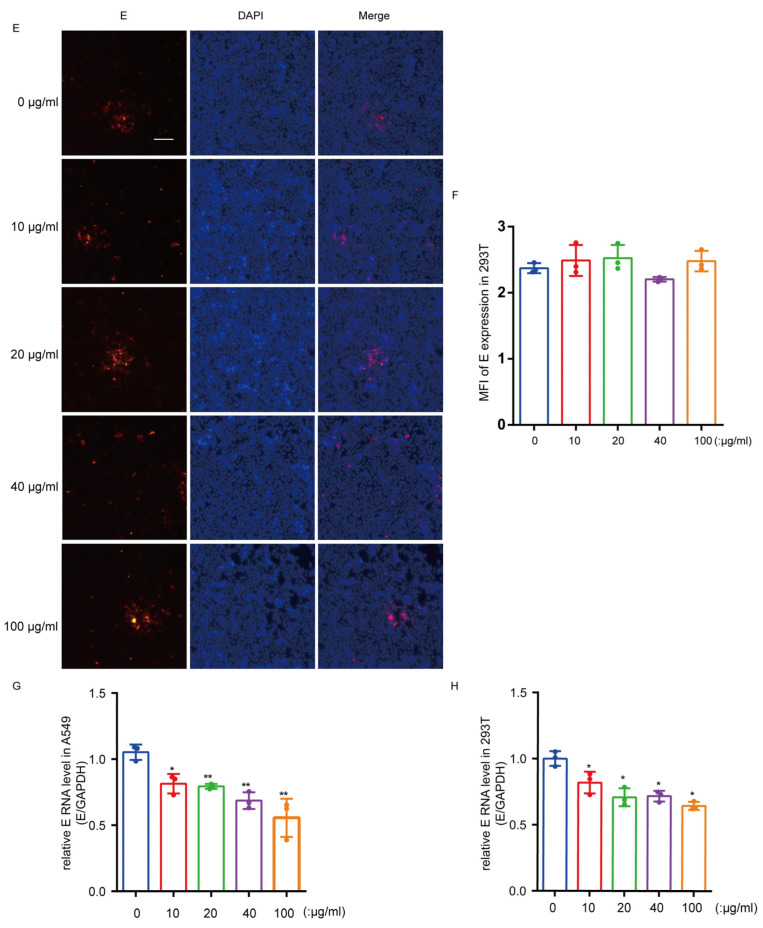
The TIM-1 ectodomain soluble protein inhibits JEV infection and entry in cells: (**A**) A549 cells were seeded onto 96-well plates and inoculated with mixture containing JEV (MOI = 0.5) and TIM-1 ECD protein with different concentrations for 36 hpi; then, the cells were fixed and subjected for fluorescence via using mouse anti-E antibody (red). The nuclei were stained with DAPI. Scale bars, 50 μm. (**C**) 293T cells were transfected with TIM-1 plasmids for 24 h. Then, the cells were infected with mixture containing JEV (MOI = 0.5) and TIM-1 ECD protein with different concentrations and subjected to IFA. Scale bars, 50 μm. (**B**,**D**) The relative red fluorescent intensity among the group of JEV E in A549 (**B**) and 293T (**D**) cells were analyzed by using ImageJ software. In addition, histograms were generated via GraphPad Prism 6.0 software. (**E**) The methods were performed in 293T cells in the same manner as in the (**A**,**C**). Scale bars, 50 μm. (**F**) The relative infection ratios were calculated using the same procedures as in (**B**,**D**). (**G**,**H**) A549 cells (**G**) and 293T cells transfected with TIM-1 plasmid (**H**) were infected with mixture containing JEV (MOI = 10) and TIM-1 ECD protein at 37 °C for 1 h and shifted into 4 °C for another 1 h and then incubated for 45 min at 37 °C. The samples were then harvested for qPCR. All data were expressed as the mean ± SD of three independent experiments. * *p* < 0.05, ** *p* <  0.01, *** *p*  <  0.001, **** *p*  <  0.0001 (one-way ANOVA analyses). SD, standard deviation.

Given the interaction between hTIM-1 protein and JEV E protein and entry-promotion function of hTIM-1 above, we considered whether the soluble recombinant ectodomain (ECD) of hTIM-1 could impede JEV infection or entry. Recombinant soluble hTIM-1 ECD protein (amino acids, 21-296) fusion with 6×His tag was prepared. For the infection inhibition assays, the viruses were preincubated with the recombinant hTIM-1 ECD protein in different dilutions for 1 h; the cells were then infected with the mixture for 36 hpi. The immunofluorescence staining results showed that recombinant hTIM-1 protein dose-dependently reduced the infection of JEV strain NJ2008 in A549 as well as 293T (exogenous expression of hTIM-1 protein). In A549 cells, with increasing hTIM-1 protein concentration, the inhibition efficiency was significantly increased, reaching 80% at the protein concentration of 100 μg/mL (Figure 4A,B). For hTIM-1-expressing 293T cells, the concentration of hTIM-1 ECD protein at 100 μg/mL achieved about 85% inhibition efficiency (Figure 4C,D). Because 293T cells naturally express a low level of TIM-1, they could be studied as a control cell. In addition, the infection ratios have no significant change when 293T cells were infected with the mixture containing the different concentrations of TIM-1 ECD protein (Figure 4E,F). Furthermore, the hTIM-1 ECD protein could also prevent JEV from entering A549 cells and hTIM-1-expressing 293T cells, with the inhibition rate at about 50% and 40%, respectively (Figure 4G,H). Considering these results, we concluded that recombinant hTIM-1 protein inhibited JEV infection in vitro.

### 3.5. hTIM-1 Antibodies Block JEV Infection in Cells

**Figure 5:**
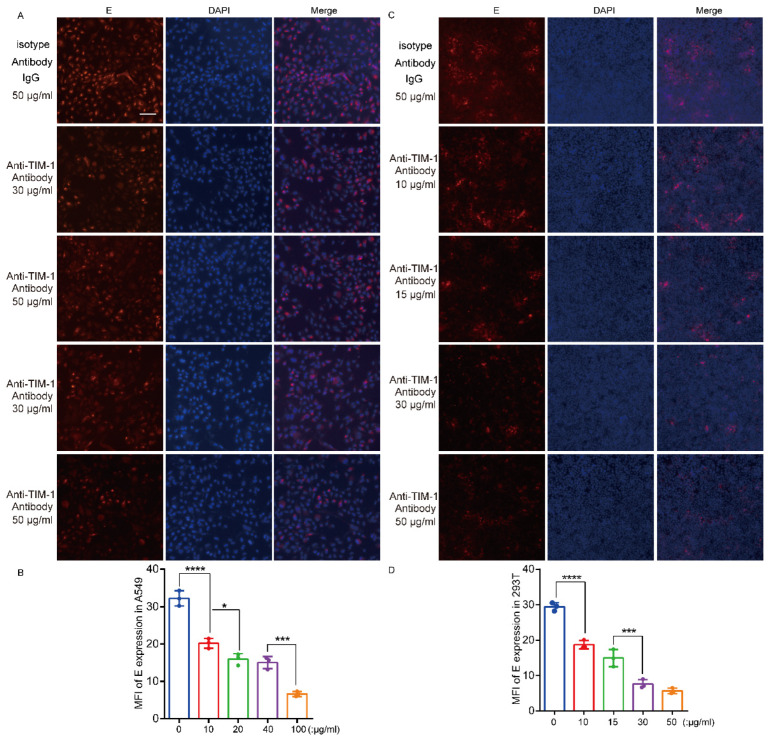

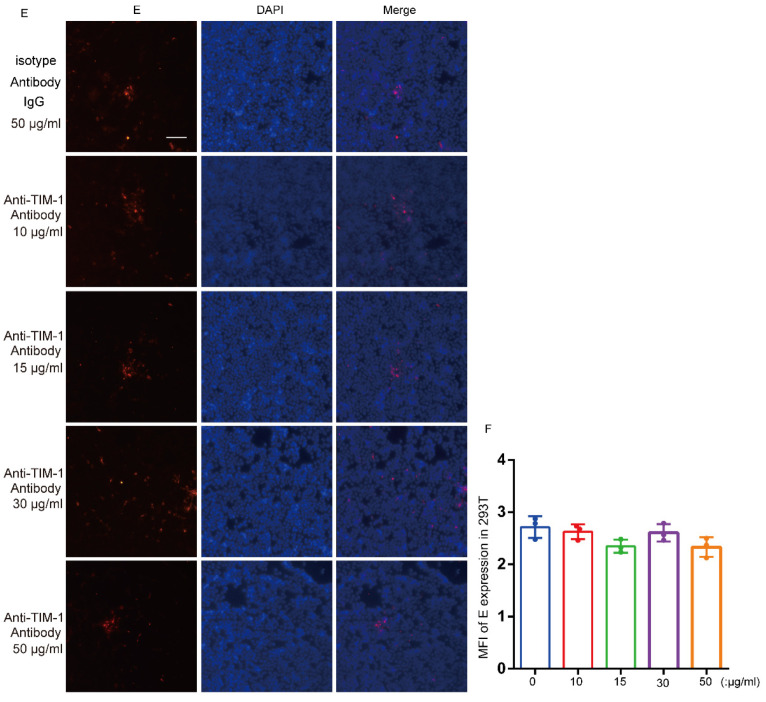
Antibodies against TIM-1 block JEV infection in cells in a dose-dependent manner: (**A**,**C**) A549 (**A**) and 293T cells transfected with TIM-1 (**C**) were seeded on 96-well plates and inoculated with 0.03 mL of medium containing different concentrations of antibodies and isotype antibody at 4 °C for 1 h before infection. Then, the cells were infected with JEV strain NJ2008 at an MOI of 0.5 at 37 °C for 36 h. The cells were fixed with 4% formaldehyde and subjected to fluorescence microscope observation. Scale bars, 50 μm. (**B**,**D**) The relative infection ratios were determined by using NJ2008 software to calculate the mean fluorescent intensity (MFI) of each image of A549 (**B**) and 293T (**D**) cells. In addition, histograms were generated via GraphPad Prism 6.0 software. (**E**) The experiments were performed in 293T cells as shown in (**A**,**C**). Scale bars, 50 μm. (**F**) The procedures described in (**B**,**D**) were used to calculate the relative infection ratios. All data were expressed as the mean ± SD of three independent experiments. * *p* < 0.05, *** *p*  <  0.001, **** *p*  <  0.0001 (one-way ANOVA analyses). SD, standard deviation.

After immunizing mice with the recombinant ectodomain protein, polyclonal antibodies against the hTIM-1 protein were prepared. We then investigated whether hTIM-1 antibodies could block the hTIM-1 protein on the cell surface to reduce JEV infection in vitro. The cells were firstly incubated with hTIM-1 antibodies at 4 °C for 1 h and then infected with JEV NJ2008 for 36 hpi. The results showed that the JEV infection efficiency decreased in a dose-dependent manner with hTIM-1 antibody treatment. When the concentration of polyclonal antibody was at 50 μg/mL, the inhibition ratio of JEV infection can reach 75% in A549 cells (Figure 5A,B). As for hTIM-1-expressing 293T cells, the inhibition rate started at 10 μg/mL and can approach about 85% at the concentration of 50 μg/mL (Figure 5C,D). As predicted, the isotype antibodies IgG at 50 μg/mL displayed no inhibitory effect on the infection. The 293T cells (non-expressing hTIM-1) also showed similar infection efficiency with different concentrations of hTIM-1 antibodies (Figure 5E,F). Therefore, these results conclusively showed that the hTIM-1 polyclonal antibodies inhibited JEV infection in vitro.

## 4. Discussion

Many studies have demonstrated that the PS-hTIM-1 protein binding enhances the infection of numerous envelope viruses, which include EBOV, Marburg virus (MARV), DENV, yellow fever virus, JEV, Chikungunya virus (CHIKV), tick-borne encephalitis virus (TBEV), and so on [37,38,39]. Some evidence indicated that hTIM-1 just binds to virion-associated PS and does not need viral protein [27,28,40]. However, hTIM-1, hTIM-3, and hTIM-4 proteins in the TIM family have the PS binding sites, but their capacity promoting flavivirus infection differs [26]. In addition, our previous study also revealed that hTIM-1 protein different variants V1, V2, and V3 possess the same PS binding residues, while only hTIM-1 V2 can effectively enhance JEV infection. In addition, this entry-promotion function of hTIM-1 is PS-dependent [41]. Therefore, it is necessary to investigate more factors that mediate JEV infection in addition to the PS–TIM-1 interaction.

Preferable usage of hTIM-1, not other PS receptors by flaviviruses and filoviruses, indicated that hTIM-1 protein may not only promote virus infection with the virion-associated PS. Later, more evidence indicated that hTIM-1 is an authentic DENV entry receptor and hTIM-1 can also interact with ZIKV E protein to promote ZIKV entry [31,32]. In addition, hTIM-1 protein can interact with EBOV GP protein and PS on the membrane to promote viral entry. So, hTIM-1 may not only be an attachment factor for JEV infection. In addition, the in vitro binding affinity studies indicated that the role of GP protein to mediate EBOV entry appears to be more important than that by PS [30]. Therefore, we speculated that hTIM-1 protein can act as a dual-attachment receptor, recruiting JEV to membrane surface by interacting with JEV E protein and PS on the viral surface. Our result of Co-IP and GST pull-down indicated that hTIM-1 can directly interact with JEV E protein.

Our results also showed TIM-1 N114 and D115 were important to the interaction between hTIM-1 and JEV E protein. When amino acids N and D at position 114 and 115 were mutated into A, their interaction was significantly weakened. Therefore, our results suggested that TIM-1 plays a dual-attachment role in promoting JEV infection, involving the TIM-1-PS binding and the interaction between TIM-1 and E protein. During the early stage of infection, the PS on the virus membrane is relatively low, and the protein–protein interaction prevails over intermolecular forces, making the interaction between TIM-1 and the E protein the primary driver of JEV infection. Consistent with our findings, previous studies have demonstrated that the mutants of hTIM-1 (N114A and D115A) show reduced ability to facilitate cellular entry of Ebola pseudovirus due to the inability of binding of viral PS or GP to the mutant hTIM-1 receptor [30]. In addition, K63 ubiquitination of lysine residues is known to mediate target protein endocytosis, and the lysine residue at position 38 of the E protein is highly conserved among flaviviruses. When this amino acid is mutated to arginine, their interaction is significantly impaired. Additionally, evidence suggests that K63-linked ubiquitination on the K38 residue of E efficiently facilitates virus attachment to host receptors, partly mediated by hTIM-1 [32].

Our findings indicated that hTIM-1 and JEV could internalize into cells and undergo co-transportation to early and late endosomes, suggesting that hTIM-1 may assist in JEV intracellular transport. STAM-1, a component of the ESCRT-0 complex involved in intracellular trafficking of ubiquitinated cargo, interacts with hTIM-1 and is necessary for DENV infection [31]. Therefore, it would be worthwhile to investigate the potential partners involved in the cytoplasmic trafficking of hTIM-1 and JEV. The transcriptomic analysis will offer us some more optional molecular chaperones at different stages of JEV infection.

Despite the presence of several PS binding receptors or other attachment factors on the surface of the A549 cell membrane, recombinant soluble hTIM-1 protein could effectively inhibit JEV entry or infection, with an inhibition rate exceeding 70%. This indicates that the hTIM-1 receptor on the membrane plays a dominant role in promoting JEV infection. Antibodies against hTIM-1 protein also exhibit significant inhibitory effects on JEV infection. It has been reported that loss of hTIM-1 expression decreased overall mortality and delayed time to death of those mice that did succumb when challenged with EBOV GP/rVSV [42]. Additionally, hTIM-1 deficiency decreases viral tissue load and pathogenicity in immunocompetent mice [37]. Recombinant TIM-1 ECD protein represents a potential therapeutic avenue for Ebola virus and its mutated species [43]. Thus, hTIM-1 is a promising target for antiviral development. Further studies on mice assays are warranted to determine whether the TIM-1 antibodies could reduce the virus loads in vivo and become a promising anti-JEV target molecule.

hTIM-1 protein expressed in a broad range of epithelial cells [19]. It is tempting to speculate that hTIM-1 could interact with envelope protein and PS as a primary dual-attachment viral receptor in the skin during mosquito bites, potentially playing a role in virus transmission.

In conclusion, we firstly identified the interaction between hTIM-1 and JEV E protein and the crucial residues contributing to their interaction. Targeting their interaction interface could be a promising intervention strategy to JEV infection. Recombinant hTIM-1 protein and antibody effectively inhibit JEV infection in vitro, providing valuable insights for the development of a JEV antiviral target. Therefore, our findings pave the way for exploring potential therapeutic strategies against JEV.

## Figures and Tables

**Table 1:** Primers for RT-qPCR.

Primer Name	Primer Sequence
hTIM-1-F	5′-AACTGTCTCTACCTTTGTTCCTCC-3′
hTIM-1-R	5′-GTTCTCTCCTTATTGCTCCCTG-3′
JEV(NJ2008/SA14-14-2)-E-F	5′-GGCAAACGACAAACCAACATT-3′
JEV(NJ2008/SA14-14-2)-E-R	5′-ATCAGCTCGCTTCTCGTTGTG-3′
JEV(HEN0701)-E-F	5′-AGAGATTCCTACTCTCGACATGC-3′
JEV(HEN0701)-E-R	5′-GCCCACAGTATCCTGCAACC-3′
GAPDH-F	5′-GAGTCAACGGATTTGGTCGT-3′
GAPDH-R	5′-GACAAGCTTCCCGTTCTCAG-3′

## Data Availability

The data presented in this study are available upon request from the corresponding author.
